# Antifungal resistance: from classical mechanisms to the expanding role of acetyltransferases

**DOI:** 10.1186/s12929-026-01273-8

**Published:** 2026-07-02

**Authors:** Silvia Maria Cordeiro Werneck, Ludmila Gouveia-Eufrasio, Gustavo José Cota de Freitas, Daniel Santana de Carvalho, Isabela da Costa César, Luana Rossato, Rafael Wesley Bastos, Nalu Teixeira Aguiar Peres, Daniel Assis Santos

**Affiliations:** 1 Department of Microbiology, Institute of Biological Sciences, Universidade Federal de Minas Gerais, Av. Pres. Antônio Carlos, 6627, Pampulha, Belo Horizonte, MG 31270-901 Brazil; 2 Department of Pharmaceutical Products, Pharmacy Faculty, Universidade Federal de Minas Gerais, Av. Pres. Antônio Carlos, 6627, Pampulha, Belo Horizonte, MG 31270-901 Brazil; 3 Postgraduate Program in Biodiversity and Evolution, Institute of Biology, Universidade Federal da Bahia, Av. Milton Santos, Ondina, Salvador, BA 40170-110 Brazil; 4 Health Sciences Research Laboratory, Universidade Federal de Grande Dourados, R. João Rosa Góes, 1761, Vila Progresso, Dourados, MS 79825-070 Brazil; 5 Department of Microbiology and Parasitology, Center for Biosciences, Universidade Federal do Rio Grande do Norte, Av. Sen. Salgado Filho, 30000, Lagoa Nova, Natal, RN 59064-741 Brazil; 6 Brazilian National Institute of Science and Technology in Human Pathogenic Fungi–INCT Funvir, R. da Reitoria, 374, Butantã, São Paulo, SP 05508-220 Brazil

**Keywords:** Acetyltransferase, Antifungal resistance, Acetylation, Fluconazole inactivation

## Abstract

Antifungal resistance represents a growing global health challenge, driven by limited therapeutic options, inadequate use of both clinical and environmental drugs, and the remarkable adaptive capacity of fungal pathogens. While resistance has classically been attributed to genetic alterations affecting drug targets and efflux systems, accumulating evidence indicates that regulatory mechanisms play a critical role in shaping antifungal susceptibility. This review highlights that acetyltransferases constitute important and active components of antifungal resistance, operating through multiple integrated mechanisms. Notably, recent studies have extended this framework by identifying the direct enzymatic inactivation of antifungal agents by acetyltransferases, which emerges as an additional resistance mechanism and establishes a functional parallel with antibiotic acetylation in bacteria. These findings highlight acetyltransferases as clinically relevant drivers of antifungal resistance and suggest that targeting these enzymes may enhance the efficacy of existing antifungal therapies and reduce treatment failure.

## Introduction

Infections caused by fungi exert a huge impact on global health, affecting more than 6 million individuals with invasive fungal infections worldwide. They are estimated to cause approximately 2.5 million deaths annually, representing a substantial threat to public health [1]. Among the most common pathogens, *Candida albicans*, *Cryptococcus neoformans*, *Candida auris*, and *Aspergillus fumigatus* have been included in the critical group of priority fungal pathogens list, recently published by the World Health Organization [2].

The limited number of fungus-specific therapeutic targets leads to a highly constrained antifungal drug repertoire that is largely confined to the azole, polyene, and echinocandin classes. Azoles obstruct ergosterol biosynthesis by inhibiting 14-α-demethylase (Erg11/Cyp51), inducing the toxic accumulation of sterols produced by Δ−5,6-desaturase (Erg3); polyenes, in addition to binding to ergosterol in the plasma membrane where they form large pores that disrupt cellular function, also extract this sterol from lipid bilayers to form aggregates that destabilize the membrane, a mechanism called the sterol sponge model; echinocandins interfere with the β−1,3-D-glucan synthase, a multi-component enzyme complex composed of an intracellular regulatory Rho GTPase and a transmembrane catalytic Fks subunit, which drives the elongation of the β-glucan chain through successive glucose incorporation. Echinocandins compromise the integrity of the fungal cell wall by inhibiting the catalytic subunit Fks of β−1,3-glucan synthase, encoded by the paralogous genes *FKS1*, *FKS2*, and *FKS3*. Evidence indicates that the affinity of this interaction is influenced by the sphingolipid composition in the glucan synthase microenvironment, suggesting that alterations in the organization of the lipid bilayer may modulate the binding of echinocandins to their target and, consequently, their antifungal activity [3–8].

The reduced availability of clinical antifungal options, together with their increasingly widespread global use, has intensified the selective pressure that favors the emergence and dissemination of resistant strains [9]. Classical antifungal resistance mechanisms include alterations in drug targets, gene amplification events, transcriptional regulation of sterol biosynthesis and efflux pumps, as well as activation of stress-response pathways [10–13].

Therefore, genetic mechanisms and epigenetic regulation, including chromatin remodeling and histone modifications, are increasingly being implicated in modulating antifungal susceptibility [14, 15]. Epigenetic processes can operate through RNA-based systems or through chromatin-dependent mechanisms. In the chromatin context, structural and chemical alterations modulate the accessibility of transcription factors to specific genomic regions, thereby redefining global patterns of gene expression [14]. Histone acetylation stands out as one of the most extensively characterized modifications, exerting a direct influence on transcriptional plasticity and on the mutational landscape of fungal cells, which favors adaptation and the emergence of drug-resistant phenotypes across different pathogens [15–17]. This post-translational modification involves the reversible acetylation of lysine residues located in the amino-terminal tails of histones. This process is catalyzed by histone acetyltransferases (HATs) and histone deacetylases (HDACs), which together control the dynamic states of chromatin often associated with responses to stress [18–21].

Further emerging evidence suggests that acetyltransferases may directly inactivate antifungal agents, challenging current resistance paradigms [22]. As the genetic and molecular bases of antifungal resistance have already been extensively examined in recent studies, this article focuses on the main antifungal resistance mechanisms involving acetyltransferases, within the framework of epigenetic regulation and through additional chromatin-independent functions [22–25].

**Fig. 1:**
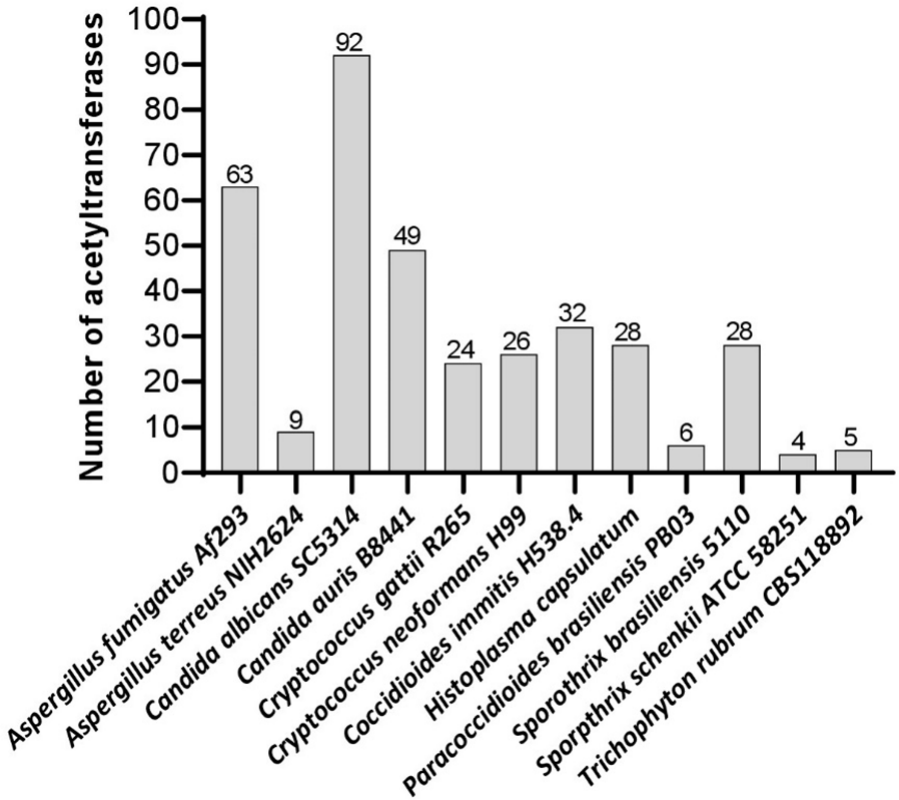
Number of acetyltransferases in the main fungal pathogens. Representation of the number of acetyltransferases genes found in current annotation of the genomes of the main fungal pathogens

Protein acetylation is a widely conserved process in fungi that is primarily associated with essential biological functions, such as the regulation of gene expression, DNA repair, metabolism, and intracellular signaling [26, 27]. This functional conservation is reflected in the widespread evolutionary preservation of acetyltransferases across major fungal pathogens, as shown in Fig. 1, based on searches of publicly available fungal databases (*Candida albicans* SC5314 [28]; *Candida auris* B8441 [29]; *Cryptococcus neoformans* var. grubii H99 [30] *Aspergillus fumigatus* Af293 [31]; *Aspergillus terreus* NIH2624 [32]; *Sporothrix brasiliensis* 5110 [33]; *Sporothrix schenckii* ATCC 58251 [34]; *Histoplasma capsulatum* G186AR [35]; *Coccidioides immitis* H538.4 [36]; *Paracoccidioides brasiliensis* Pb03 [37]; *Trichophyton rubrum CBS* 118892 [38]). Comparative analyses indicate that fungi share multiple classes of acetyltransferases, including N-terminal acetyltransferases (NATs), O-terminal acetyltransferases (OATs), HATs, alcohol acetyltransferases (AATs), S-acetyltransferases/palmitoyltransferases (PATs), and N-myristoyltransferases (NMTs) [39]. These acetyltransferase families exhibit substantial structural and functional diversity, fulfilling distinct roles in the regulation of several fungal cellular processes.

HATs are particularly notable among these conserved groups for their direct association with antifungal resistance mechanisms, largely owing to their ability to modulate adaptive transcriptional programmes [11, 40]. HATs can be classified into families based on sequence homology within the HAT domain present in the catalytic subunit [39, 41]. Prominent families include the Gcn5 (General Control Non-Repressible 5)/PCAF (p300/CBP-associated factor) family; the fungal-specific KAT11 family, whose principal representative is the acetyltransferase Rtt109; the MYST family and the p300/CBP family. However, among these families, only Gcn5 and Rtt109 were associated with antifungal resistance, as shown in Table 1 [39, 41–46].

**Table 1:** Main HATs conserved in fungi and involved in antifungal resistance

Family	(Catalytic subunit)/HAT complex/Core components	Associated resistance	Fungi	References
Gcn5	Gcn5/SAGA/Ada2-Spt	Deletion of Gcn5 increases susceptibility to echinocandin and fluconazole	*C. albicans*	[47–49]
		Ada2 deletion increases sensitivity to azoles, polyenes, and echinocandins	*C. glabrata* (*N. glabratum*)	[50]
		Gcn5 induces transcription of *FKS*, which encodes 1,3-glucan synthase. Deletion of *GCN5* alters cell wall composition and increases susceptibility to echinocandins	*C. albicans,* *C. krusei,* *C. tropicalis*	[47, 49, 51]
		Gcn5 activates transcription of the *ERG11* gene. Deletion of *GCN5* reduces the expression of *ERG11, ERG3*, and *ERG1*, and drug efflux pumps, increasing sensitivity to azoles, echinocandins, and polyenes	*C. auris*	[52]
		Gcn5 activates transcription of the efflux pumps *MDR1* and *CDR1* genes. Deletion of *GCN5* or *ADA2* decreases transcription of efflux pumps coding genes	*C. albicans,* *C. gattii,* *C. neoformans*	[22, 51, 53–57]
	Hat1/NuB4/Hat1-Hat2	Hat1 deletion increases susceptibility to echinocandins	*C. albicans*	[58]
		Hat1 deletion increases resistance to azoles. The mechanism remains undefined	*C. albicans*	[59]
	Hat2/NuB4/Hat1-Hat2	Hat2 deletion increases resistance to azoles. The mechanism remains undefined	*C. albicans*	[59]
Rtt109	Rtt109/Histone chaperones Vps75 and Asf1/Chaperones Vps75 and Asf1	Rtt109 maintains DNA integrity. Deletion causes DNA damage and increases susceptibility to echinocandins and 5-fluorocytosine	*C. albicans*	[60–62]
		Deletion of Rtt109 decreases *CDR1* and *MDR1* expression and increases susceptibility to azoles	*C. albicans,* *C. tropicalis*	[63]
		Rtt109 modulates morphogenesis, virulence, and signaling pathways leading to antifungal resistance	*A. flavus,* *A. fumigatus*	[46]

**Fig. 2:**
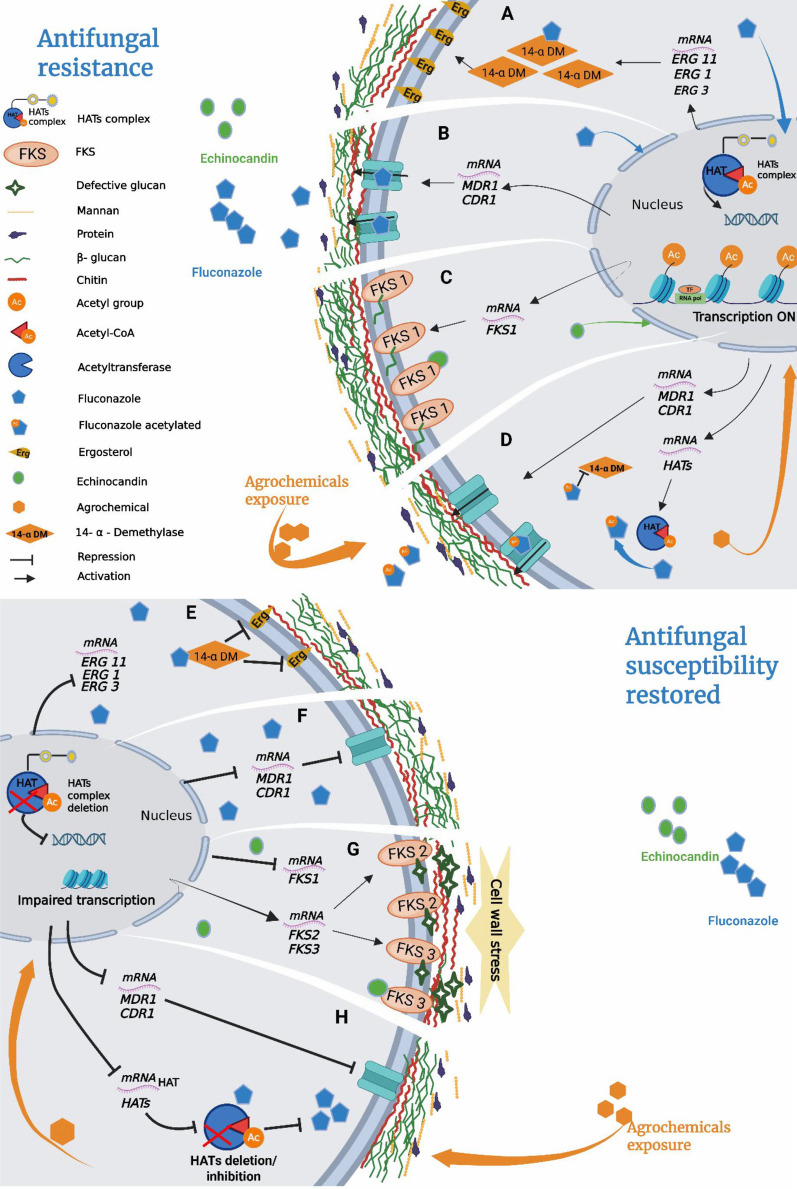
Acetyltransferases as active regulatory centers, integrating epigenetic control and metabolic detoxification of drugs. HATs promote the overexpression of the genes *ERG11*, *ERG1, ERG3* coding the 14- α-demethylase enzyme, target of azoles (**A**); *MDR1* and *CDR1*, coding efflux pump genes (**B**); and the genes involved in cell wall biosynthesis, such as glucan synthases FKS (**C**), promoting an antifungal resistance phenotype. Exposure to agrochemicals increases Hat expression and pharmacological alteration of fluconazole by adding and acetyl group by these enzymes, preventing its binding to the 14-α-demethylase enzyme, in addition to increasing the expression of efflux pumps (**D**), contributing to an antifungal resistance. Pharmacological inhibition or gene deletion of HATs reduces the expression of 14-α- demethylase genes and membrane ergosterol levels (**E**); reduces the expression of *MDR1* and *CDR1* efflux pump genes, increasing intracellular antifungal levels (**F**); reduces the expression of cell wall biosynthesis genes (1,3-glucan synthase – *FKS1*), induces the compensatory upregulation of *FKS2* and *FKS3*, promoting a distinct distribution of glucan or an aberrant integration of newly synthesized glucan. These changes, associated with increased chitin levels, alter cell wall stability and increase susceptibility to caspofungin (**G**); inhibit fluconazole acetylation and efflux pumps levels, restoring its activity (**H**), also contributing to restoring antifungal susceptibility

In general, these acetyltransferases function as catalytic components within multiprotein complexes that include regulatory subunits and transcriptional co-activators (Table 1). Within these complexes, HATs catalyze the transfer of acetyl groups from acetyl-coenzyme A (acetyl-CoA) donor to specific lysine residues on histones. This modification promotes chromatin relaxation, thereby increasing DNA accessibility to transcription factors, modulating the expression of target genes. In the context of antifungal resistance, such chromatin remodeling facilitates the regulation of critical genes involved in adaptation to pharmacological stress, triggering mechanisms that establish a resistant phenotype (Fig. 2) [39, 46, 64].

## Classic mechanisms of antifungal resistance

Antifungal resistance is primarily linked to mutations or overexpression of the gene encoding the drug target. These mutations can either alter the protein sequence, thereby impairing drug inhibition, or change the binding of transcription factors in the promoter region. Azole resistance is predominantly related to genetic changes affecting the genes that encode the azole target enzyme, lanosterol 14α-demethylase, known as *ERG11* in yeasts and *CYP51* in molds. In *A. fumigatus*, which expresses two paralogous lanosterol 14α-demethylase isoenzymes*, cyp51A* and *cyp51B*, alterations in the molecular target are the most documented resistance mechanism, with several distinct mutational variants being described in *cyp51A* [65, 66]. Surveillance studies conducted in the Netherlands, have linked the emergence and environmental spread of azole-resistant *A. fumigatus* isolates to tandem-repeat insertions in the *cyp51A* promoter associated with the amino acid substitutions TR34/L98H and TR46/Y121F/T289A [67–69]. Importantly, these resistance-associated genotypes have been identified both in agricultural environments subjected to azole fungicide pressure and in antifungal-naive hospitalized patients, reinforcing the hypothesis that environmentally selected resistant strains can subsequently enter the clinical setting [67, 68]. Genomic profiling of *A. fumigatus* isolates from the United States of America supports the hypothesis that dissemination of the TR34/L98H resistance genotype resulted from a common ancestral lineage that subsequently spread through genetic recombination with susceptible strains [70]. Distinct amino acid substitutions in Erg11 have been described in clinical isolates of *C. albicans* [12, 71]. Structural analyses have shown that several of these Erg11 substitutions, including R467K and G464S, are located near the heme-binding site and directly interfere with azole binding [12, 72]. ERG11 point mutations also exert a stronger phenotypic impact in haploid fungi, such as *C. glabrata* [73, 74]. In contrast, only a limited number of ERG11 mutations have been identified in azole-resistant clinical isolates of *C. neoformans*, including Y145F [75, 76]. Mutations in ERG11 have also been associated with reduced azole susceptibility in the emerging multidrug-resistant pathogen *C. auris* [77–79]. In *A. fumigatus*, the enzyme 3-hydroxy-3-methylglutaryl-coenzyme A (HMG-CoA) reductase catalyzes the first committed step in ergosterol biosynthesis. Mutations in *hmg1*, such as those that alter residues in the predicted sterol-sensing domain (P309L and I412T), have recently emerged as additional molecular determinants associated with triazole resistance in clinical isolates of A. fumigatus [80, 81].

Beyond structural alterations, increased *ERG11* expression has been associated with gene amplification and gain-of-function mutations in the Zn^2^⁺–Cys₆ transcription factor Upc2, a regulator present as a single copy in *C. albicans*, and as two functional homologues, Upc2a and Upc2b, in *C. glabrata* [24, 25, 82, 83]. In *Aspergillus*, the loss of AtrR, a fungal-specific Zn2-Cys6 transcriptional regulator that controls the expression of *CYP51A* and *CDR1B*, markedly increases susceptibility to azoles, highlighting its role as a key regulator of ergosterol biosynthesis and drug efflux pathways [84]. In *C. glabrata*, activation of the zinc cluster transcription factor, Pdr1 (pleiotropic drug resistance), stimulates *CDR1* transcription, representing a central axis of azole resistance [24, 85, 86]. The loss of function of NctA, a transcription factor belonging to the evolutionarily conserved CBF/NF-κB family, in *A. fumigatus*, confers resistance to different classes of antifungals, including azoles and amphotericin B. This phenotype has been associated with cell wall remodeling that limits the accessibility of ergosterol from the plasma membrane, reducing the effectiveness of compounds that depend on this molecule to exert their antifungal activity [87].

In parallel, alterations in *ERG3*, which encodes an enzyme Δ−5,6-desaturase, essential for episterol conversion within the ergosterol biosynthetic pathway, contribute to azole resistance by preventing the formation of toxic sterol intermediates normally generated during exposure to these antifungal agents [24, 25].Concerning echinocandin resistance, mutations in the *FKS1* gene, encoding a catalytic subunit of the 1,3-β-glucan synthase complex, have been identified in *C. albicans*, *Cryptococcus* spp., and *Aspergillus* spp., whereas mutations in *FKS2* contribute specifically to resistance in *C. glabrata* [88, 89].

Another well-established underlying mechanism for antifungal resistance is efflux pumps. Cellular efflux is primarily mediated by two major classes of transport proteins: the ATP-binding cassette (ABC) transporters, which are energized by ATP hydrolysis, and the major facilitator superfamily (MFS) transporters**,** which function through mechanisms coupled to electrochemical gradients. Unlike echinocandins and polyenes, azole resistance is related to increased expression of efflux pumps, thus reducing the intracellular concentration of antimicrobials (Fig. 2B) [90]. *Aspergillus fumigatus* upregulates the ABC transporter AtrF in response to itraconazole exposure, whereas *C. neoformans* utilizes the ABC transporter Afr1 to export fluconazole [13, 91, 92]. In *Cryptococcus neoformans*, disruption of *CnAFR1* in a previously resistant isolate resulted in a marked increase in fluconazole susceptibility in the *CnAFR1* knockout mutant. Conversely, genetic complementation through reintroduction of *CnAFR1* restored the resistant phenotype, demonstrating a direct contribution of this transporter to fluconazole resistance [13]. In *Candida albicans*, the overexpression of the ABC transporters such as *CDR1* and *CDR2* (Candida drug resistance 1 and 2), and MFS transporter *MDR1* (multidrug resistance 1) is frequently observed in fluconazole-resistant strains, frequently driven by activating mutations in the transcriptional regulators *TAC1* and *MRR1* [93–95]. Evidence from *A. fumigatus* also indicates that azole-resistant isolates exhibit increased expression of multiple ABC transporters, including *MDR1* and *CDR1B*, transporters that has been directly demonstrated to contribute to resistance [96, 97]. In *C. auris*, fluconazole resistance results from gain-of-function mutations in *TAC1B*, which directly enhance CDR pump expression [98–100]. Clade III-specific *MRR1A* variants, such as N647T, also positively modulate *C. auris* MFS-type transporter gene, *MDR1,* dependent resistance [101].

Furthermore, chromosomal alterations, including loss of heterozygosity (LOH), aneuploidy, and increased copy numbers at loci encoding efflux regulators or transporters, amplify the drug efflux capacity, particularly in *C. glabrata, C. albicans*, and *C. neoformans* [76, 102–105]. In *Candida albicans*, azole resistance has been correlated with the formation of the chromosome 5 left-arm isochromosome (i(5L)) [102].This aneuploid rearrangement promotes increased expression of *ERG11* and of transcriptional regulator of efflux pumps *TAC1*, resulting in the coordinated enhancement of multiple resistance determinants [106]. Exposure of *C. albicans* to hydroxyurea or tunicamycin selects for chromosome 2 trisomy, a genomic alteration related to increased tolerance to caspofungin [107, 108]. In *Cryptococcus neoformans*, fluconazole resistance has been associated with chromosome 1 disomy, a genomic alteration that promotes increased expression of both *ERG11* and the azole transporter *AFR1*, particularly during antifungal-induced stress [104]. Loss of heterozygosity (LOH) also represents an important genomic mechanism contributing to antifungal resistance. In Candida albicans, by eliminating the wild-type allele while retaining a resistance-associated variant, LOH can promote increased *MDR1* expression, thereby reducing intracellular concentrations of azole antifungals [109]. Gene duplication events also contribute to antifungal resistance, as exemplified by amplification of *MDR1* in *Candida albicans* and *C. dubliniensi* [110, 111]. This genomic plasticity allows fungal pathogens to adapt to environmental and therapeutic pressures.

## Direct and indirect roles of acetyltransferases in antifungal resistance

### Acetyltransferases and the epigenetic regulation of antifungal resistance

Notably, HATs such as Gcn5 and Rtt109 play a central role in orchestrating transcriptional programs that influence ergosterol metabolism, cell wall remodeling and drug efflux and integrity pathways, and, thereby enabling fungi to adapt to the antimicrobial stress (Fig. 2A–C) [47, 48, 52, 112]. Gcn5 and Ada2 are subunits of the Spt–Ada–Gcn5 acetyltransferase (SAGA) complex, a central chromatin-modifying assembly component that governs histone H3K9 acetylation and coordinates the transcriptional regulation of a wide range of genes [51]. In *C. albicans*, deletion of *GCN5* increases sensitivity to echinocandins, and this phenotype occurs independently of elevated levels of reactive oxygen species [47]. Similarly, deletion of *ADA2* in *C. glabrata* (*Nakaseomyces glabratum*) results in susceptibility to azoles, polyenes, and echinocandins, likely due to altered cell wall integrity, since genes encoding ABC transporters remaining unaltered [50]. Furthermore, inhibition of *GCN5* in *C. glabrata* increased fluconazole susceptibility, while markedly suppressing the acquisition of resistance during drug exposure, in part by preventing the emergence of gain-of-function alleles in *PDR1* (Pleiotropic Drug Resistance 1) a transcriptional regulator involved in multidrug resistance [48, 112].

The fungal-specific acetyltransferase Rtt109 expands this regulatory landscape by integrating processes such as chromatin assembly, DNA repair, virulence, and antifungal tolerance. Rtt109-mediated acetylation of the histone H3 (H3K56ac) is essential for replication-coupled nucleosome assembly and genomic stability. Its activity requires the formation of a complex with the histone chaperones Vps75 and Asf1 [60, 61, 113]. In *C. albicans*, loss of *RTT109* triggers DNA damage signaling, increases susceptibility to echinocandins, probably due to oxidative stress, and renders cells hypersensitive to antifungals targeting DNA synthesis, such as 5-fluorocytosine [60–62]. Antifungal exposure induces replicative stress and genomic instability, and therefore, disruption of Rtt109-dependent acetylation compromises chromatin integrity, turning fungal cells more susceptible to antifungal agents. This supports a direct link between acetyltransferase-mediated epigenetic regulation and drug tolerance [61]. In filamentous fungi, Rtt109 also modulates morphogenesis and secondary metabolism, including aflatoxin biosynthesis in *Aspergillus,* which are functionally associated with increased susceptibility to antifungal agents [46].

Pharmacological inhibition reinforces the role of acetyltransferases in antifungal tolerance. CPTH2, a HAT inhibitor that modulates the Gcn5 network, selectively kills CTG-clade *Candida* species, including *C. albicans*, while sparing non-CTG species, like *C. glabrata*, *C. kefyr,* and *C. lipolytica,* suggesting that one or more histone acetyltransferases are essential for fungal survival under host-relevant stress [114]. Notably, CPTH2-mediated antifungal activity occurs independently of direct Gcn5 inhibition and without a global loss of histone H3 acetylation. It is suggested that additional acetyltransferases, possibly the fungal-specific Rtt109 or the NAT10 orthologue, contribute to essential acetylation-dependent processes required for survival under stress, such as drug exposure [114].

In addition to Rtt109 and Gcn5, multiple components involved in histone H4 acetylation and chromatin assembly influence susceptibility to echinocandins and azoles. *HAT1* and *HAT2*, regulatory subunits of the NuB4 chromatin assembly, associated acetyltransferase complex involved in the acetylation and incorporation of H4–H3 histones into chromatin, contributes to caspofungin tolerance in *C. albicans*. Loss of both histone acetyltransferases, in a single and in a double mutant, leads to increased echinocandin sensitivity. In *HAT1* mutants, this phenotype is not associated with elevated intracellular levels of reactive oxygen species following exposure to caspofungin [58]. Curiously, the deletion of *HAT1* and *HAT2*, whether isolated or not, produces the opposite effect on azole susceptibility, conferring resistance to voriconazole and itraconazole. [59]. These findings indicate that Hat1-Hat2-dependent acetylation exerts opposing regulatory effects on azole and echinocandin responses, although the precise mechanism remains undefined.

Altogether, these findings reveal an integrated epigenetic architecture in which acetyltransferases operate as central nodes, coupling chromatin structure to transcriptional plasticity, morphogenesis, efflux regulation, and genome stability. Their interplay with deacetylases, RNA-based silencing, and chromatin-remodeling complexes enables fungi to recalibrate gene expression programs during antifungal exposure.

### Acetyltransferases and drug target alterations and overexpression

Alterations affecting antifungal drug targets, as well as their increased expression, represent a widely conserved mechanism underlying acquired antifungal resistance. Growing evidence indicates that epigenetic regulation, particularly mediated by acetyltransferases, plays a significant role in these resistance mechanisms (Fig. 2A–C) [47, 112].

In *Candida*, Gcn5 activates *FKS1*, which encodes the β−1,3-glucan synthase targeted by echinocandins. Thus, the increased expression of *FKS1* upon caspofungin treatment in wild-type cells explains the CSP tolerance (Fig. 2C). Deletion of *GCN5* leads to repression of *FKS1* expression while concomitantly activating compensatory pathways marked by transcriptional upregulation of *FKS2* and *FKS3* (Fig. 2G)*.* However, the enhanced glucan synthesis driven by these isoforms is insufficient to maintain caspofungin tolerance in the absence of *GCN5*. This phenotype indicates that alterations in the structure, deposition, spatial organization, or incorporation of newly synthesized glucan, together with changes in chitin composition, give rise to dysfunctional cell wall remodeling (Fig. 2G). Moreover, these structural perturbations adversely affect the transcriptional regulation of genes critical for adaptive stress responses and antifungal drug resistance, including *EFG1* and *ALS1*, thereby exacerbating cellular susceptibility to pharmacological intervention. [47, 112]. Similarly, exposure to chitosan significantly reduces antifungal resistance in caspofungin-resistant isolates of *Candida albicans*, *C. krusei* (*Pichia kudriavzevii*), and *C. tropicalis*. This is driven by transcriptional repression of *GCN5* and *ADA2*, decreased *FKS* expression, and defects in cell wall thickness and integrity. Chitosan exposure suppresses acetyltransferases and increases caspofungin efficacy, indicating it as a potential target for antifungal therapies [16, 51]. Also, Gcn5 regulates echinocandin resistance by promoting histone acetylation at the promoter sites of *CRZ1* and *CAS5* genes upon caspofungin exposure. This resistance involves epigenetic activation of the calcineurin–Cas5 signaling and regulation of genes associated with cell wall integrity. Therefore, these findings establish a direct link between chromatin acetylation and stress-responsive signaling pathways [52].

In the case of azoles, the specific target is Erg11 in yeasts and homologous to Cyp51 paralogues A and B for most filamentous fungi, Cyp51C and Cyp51D for some others species, like *Fusarium graminearum* and a subgroup of *Talaromyces* species, respectively [115–117]. Gcn5-dependent histone acetylation is essential to promote *ERG11* basal transcription, and particularly during azole exposure (Fig. 2A). Loss of *GCN5* results in hypersusceptibility to azoles, echinocandins and polyenes due to the reduced acetylation of histone H3 and the consequent suppression of the transcription of key genes in the ergosterol biosynthesis (*ERG11*, *ERG1*, *ERG3*) (Fig. 2E) and the major drug efflux pumps (*CDR1*, *SNQ2*, *MDR1*) (Fig. 2F) [52].

These findings establish drug-target alteration and overexpression as canonical mechanisms of antifungal resistance, meanwhile expanding evidence demonstrates that epigenetic regulation, particularly that mediated by acetyltransferases, plays a direct role in these resistance processes.

### Efflux pumps regulation and acetyltransferases

Increased expression of the drug efflux pump in the cell membrane is a dominant and widely conserved mechanism of azole resistance in pathogenic fungi, occurring in both clinical isolates and strains adapted to environmental pressures. Although efflux-mediated resistance has classically been attributed to genetic mutations affecting transporters or their regulators, an increasing body of evidence suggests that epigenetic control, particularly acetyltransferase-dependent chromatin regulation, constitutes a central regulatory axis governing efflux activation and antifungal resistance (Fig. 2B) [11, 40]. Histone acetylation has emerged as a key upstream factor involved in efflux pump expression. Large-scale genomic analyses of antifungal resistance networks indicate that chromatin accessibility controls the activation of multidrug transporter genes under chemical stress [11]. Lysine acetyltransferases, particularly Gcn5, enable this response by remodeling chromatin at the promoter region of stress-responsive and drug-resistance loci, thereby inducing the transcription of efflux pumps [40]. In *C. tropicalis*, functional inhibition of acetyltransferase activity has been shown to suppress the expression of major efflux genes, including *CDR1* and *MDR1*, to reduce biofilm-associated efflux capacity, and to restore azole susceptibility (Fig. 2F). Therefore, these findings establish a direct link between acetylation-dependent chromatin regulation and efflux-mediated resistance [63]. Similarly, suppression of *ADA2* by chitosan in *C. albicans* impaired *MDR1* and *CDR1* expression, thereby interfering with the regulation of the corresponding efflux pumps [16]. Additionally, disruption of Ada2 increased fluconazole sensitivity in *C. albicans*, accompanied by reduced H3K9 acetylation at the *MDR1* promoter and its expression in response to fluconazole [53]. Efflux-mediated resistance is an adaptive strategy employed by *C. neoformans* and *C. gattii*. Resistant isolates exhibited increased expression of ABC and major facilitator superfamily (MFS) transporters, including *AFR1*, *AFR2*, *AFR3*, and *MDR1*. This leads to decreased intracellular azole concentration and stable resistance phenotypes, despite the absence of *ERG11* mutations. It is suggested that this phenotype is epigenetically regulated by acetyltransferase-dependent chromatin modifications [54–57]. Furthermore, exposure to agrochemicals induces the upregulation of *GCN5* and *NAT10*, as well as the transcriptional activation of the multidrug efflux transporter *MDR1* [22].

### Other epigenetic models

Epigenetic regulation of antifungal responses is not restricted to acetylation. Set1, for example, is a histone H3K4 methyltransferase that acts as a key epigenetic regulator of azole susceptibility in a species-dependent manner [118]. In *Candida glabrata*, Set1-mediated H3K4 methylation is dynamically enriched at genes involved in ergosterol biosynthesis, including *ERG11* and *ERG3*. Loss of *SET1* abolishes this modification, impairs azole-induced activation of *ERG* genes, and reduces intracellular ergosterol levels, thus resulting in pronounced azole susceptibility. In this species, Set1 does not regulate efflux pump expression, indicating that epigenetic control of resistance predominantly operates through modulation of *ERG* gene expression rather than drug export. In contrast, in *Saccharomyces cerevisiae*, loss of *SET1* reduces azole tolerance by compromising the expression and function of the efflux pump Pdr5, highlighting species-specific differences in the epigenetic architecture underlying antifungal resistance [118].

Alterations in histone abundance also modulate antifungal susceptibility. In *C. glabrata*, two redundant FK506-binding proteins, Fpr3 and Fpr4, act as histone chaperones, maintaining H3 and H4 levels, and functioning as negative regulators of the *PDR1* regulon under basal conditions. Loss of both proteins leads to increased histone abundance, constitutive overexpression of *PDR1*, ABC transporter genes, and fluconazole resistance [119]. In this species, fluconazole exposure induces accumulation of histones H3 and H4, suggesting that histone abundance is dynamically regulated during antifungal stress, culminating in fluconazole resistance [119].

Furthermore, chromatin remodelers have also been implicated in antifungal resistance. In *C. albicans*, the SWI/SNF chromatin remodeling complex acts as a coactivator of Mrr1, a resistance regulator involved in *MDR1* expression. Deletion of *SNF2* suppresses *MDR1* overexpression even in strains harboring gain-of-function mutations in *MRR1*, thereby reducing fluconazole resistance. [120]. In *C. glabrata*, SWI/SNF and the fungal-specific chaperone Rtt106 are required for multidrug transporter expression, thereby strengthening azole resistance [113]. Deacetylation is also required for the stability and function of Hsp90, a central regulator of stress adaptation. Inhibition of lysine deacetylases disrupts Hsp90–calcineurin interaction, blocking the induction of Hsp90-dependent stress pathways that are essential for maintaining azole resistance [121]. A similar acetylation/deacetylation-dependent regulation of Hsp90 contributes to both azole and echinocandin resistance in *Aspergillus fumigatus*, in which deacetylation of lysine 27 is required for drug tolerance [122].

Fungi also employ RNA-mediated mechanisms to develop antifungal resistance [11]. In Mucorales, RNA interference (RNAi)-mediated epimutations provide an additional, fast, and reversible mechanism of antifungal resistance by silencing drug-target mRNAs [123, 124].

## Antifungal enzymatic inactivation by acetyltransferases

Recently, a previously unrecognized antifungal resistance strategy was described in *Cryptococcus* spp., in which acetyltransferase enzymes directly inactivate fluconazole by catalyzing its conversion into *O*-acetyl-fluconazole (Fig. 2D). This mechanism expands the functional scope of these enzymes beyond chromatin regulation [22]. This modification prevents fluconazole from interacting with its target, lanosterol 14α-demethylase, thus abolishing its antifungal activity. Interestingly, exposure to agricultural fungicides induces the broad overexpression of acetyltransferase genes, including *GCN5* and *NAT10*. These enzymes not only catalyze fluconazole modification but also increase *MDR1* expression (Fig. 2D). Notably, resistant isolates do not harbor *ERG11* mutations, indicating that resistance arises through an enzymatic drug-detoxification pathway rather than alteration of the azole target. These responses correlate with the emergence of cross-resistance to fluconazole in *C. deuterogattii* and *C. neoformans* [54, 125, 126].

The clinical relevance of this mechanism is reinforced by the detection of acetylated fluconazole in the cerebrospinal fluid of patients undergoing fluconazole therapy, as well as in fungal colonies recovered during therapeutic failure. These findings suggest that environmental exposure to fungicides may prime *Cryptococcus* with a pre-adapted metabolic repertoire that compromises first-line azole-based therapies. The observation that Gcn5 and **N**AT10 inhibitors, such as curcumin**,** CPTH2, and remodelin, restore fluconazole susceptibility indicates that acetyltransferase-targeted adjuvant therapies may be an effective rational strategy to neutralize the enzymatic drug inactivation (Fig. 2H) [22, 127–129].

Importantly, this resistance mechanism is not restricted to *Cryptococcus* spp. Fluconazole acetylation has also been detected in *C. albicans*, *C. auris*, and *Trichosporon* spp., indicating that acetyltransferase-dependent drug modification is conserved across different pathogenic fungal species. This mechanism is analogous to bacterial resistance pathways, in which aminoglycosides are enzymatically acetylated by acetyltransferases, resulting in antibiotic inactivation [130–132].

By demonstrating that fungi exposed to agrochemicals can inactivate clinical antifungal agents through enzymatic modification, Gouveia-Eufrasio et al. (2026) expanded the concept of antifungal resistance beyond classical genetic and epigenetic mechanisms. From a "One Health" perspective, these findings highlight the role of environmental pressures in the metabolic reprogramming of fungi and the emergence of resistance. This reinforces the need for more rigorous monitoring of agrochemical use, as well as the development of strategies capable of neutralizing these compounds in the soil and mitigating the selective pressure exerted on environmental fungi. In the clinical context, therapeutic approaches based on the inhibition of fungal acetyltransferases emerge as promising alternatives, similar to β-lactamase inhibitors used in the management of antibacterial resistance.

## Conclusions

Antifungal resistance is a complex and dynamic phenomenon that goes beyond classical genetic mechanisms. Growing evidence highlights the role of acetyltransferases, particularly histone acetyltransferases (HATs), in fungal adaptation. By modulating chromatin accessibility, HATs reprogram gene networks that control the expression of drug targets, efflux pumps, cell wall integrity, and stress response pathways, thus allowing rapid phenotypic adaptation under antifungal pressure. The recent discovery that these enzymes can directly mediate the enzymatic modification and inactivation of antifungal agents, such as fluconazole, represents an expansion of the current antifungal resistance paradigm and reinforces the metabolic plasticity of pathogenic fungi. Although this mechanism still requires further validation and investigation by independent research groups, these findings provide new perspectives for a deeper understanding of the relationship between fungal metabolism and antifungal inactivation, as well as the prevalence and clinical relevance of this phenomenon in the context of antifungal resistance.

## Data Availability

No datasets were generated or analysed during the current study.
